# A Six-month Retrospective Study of Resources Burden by Trauma Victims in the Surgical Intensive Care Unit of a University Hospital in Pakistan

**DOI:** 10.7759/cureus.3236

**Published:** 2018-08-30

**Authors:** Muhammad Rizwan, Madiha Hashmi, Hasnain Zafar

**Affiliations:** 1 Adult Critical Care Medicine, King Faisal Specialist Hospital and Research Center, Riyadh, SAU; 2 Anaesthesiology, Aga Khan University Hospital, Karachi, PAK; 3 Surgery, Aga Khan University Hospital, Karachi, PAK

**Keywords:** intensive care, resources use, developing country, trauma

## Abstract

Introduction

Trauma is the fourth leading cause of death globally and constitutes a huge burden on limited critical care resources.

Aim

​​​​This study aimed to identify the trauma patient burden in terms of resources used in the surgical intensive care unit (SICU) of Aga Khan University Hospital in Pakistan which also included characteristics and outcomes of trauma and non-trauma patients.

​​​Methods

​​​We retrospectively reviewed all patient data for adult patients (>16 years old) admitted to the SICU from July through December 2014.

Results

Of 141 SICU cases included in our study period, 32 (22.7%) trauma patients were identified. On further stratification of trauma patients, road traffic injuries (43.8%), gunshot injuries (43.8%), and blast injuries (6.3%) were the most common, and about 73% of all trauma patients underwent emergency surgical interventions, comprising a huge burden on all resources. The average age of the trauma patients was significantly lower than non-trauma patients (36 years ± 13 vs. 49 years ± 19; p < 0.01). The male-to-female ratio was 7:1 in trauma cases and 2:1 in non-trauma cases (p = 0.019).

There was no statistically significant difference in mortality (31.3% vs. 42.2% p > 0.05) and median length of stay [Median (interquartile range), 5(8) vs. 4(7); p > 0.05] between trauma and non-trauma patients.

​​​​​​Conclusions

Trauma constitutes a significant burden in terms of resources used for the SICU of the Aga Khan University, Pakistan. Trauma victims are predominantly young men in whom gunshot injuries are as common as road traffic injuries. Emergency surgical interventions comprise the largest draw on resources, followed by use of blood products, radiological, and laboratory investigations.

## Introduction

Trauma is a worldwide public health problem and the fourth leading cause of death in the western world, especially in young people (i.e., those under 40 years old). Severe trauma cases, although small in number, may need urgent lifesaving interventions, damage control surgical interventions or life-sustaining organ support in an intensive care unit (ICU), consuming significant resources and causing a huge economic burden to the patient, healthcare system, and society [1].

The 48-bed state-of-the-art Emergency Department at Aga Khan University Hospital is a World Health Organization Collaborating Center for Trauma and handles around 60,000 patients annually. The surgical ICU (SICU) has a capacity of seven beds, and major trauma victims requiring close monitoring or organ support with or without surgical intervention comprise 30% of surgical admissions.

We conducted a retrospective study from July to December 2014 to determine the characteristics of patients admitted to the SICU secondary to traumatic injuries and highlighted the critical therapeutic interventions, advanced radiological procedures, and laboratory and blood products utilized during their stay. This study also looked at the type of trauma, the source of admission, length of stay, and outcomes.

The impact of trauma on our limited resources of time, critical therapeutic interventions, advanced radiological interventions, laboratory workload, and blood product utilization has not been documented in Pakistan. This study emphasizes the resources required to safely and efficiently manage critically ill trauma patients and can be used to plan future trauma management facilities and improve the care of major trauma patients in our country in a comprehensive way.

## Materials and methods

This study was conducted retrospectively in period already mentioned. After receiving the Ethical Review Committee’s exemption for approval, all patients over age 16 years admitted during the study period were included.

Patient medical records were reviewed for age, gender, diagnosis, the source of admission, length of stay, and outcome.

The above record also confirmed by the patient care inquiry (PCI) system on a remote desktop program on the intranet maintained by the Health Information Management Service. The PCI system further maintains data that includes a source of admission, types of trauma, and other resources used by patients in the SICU like surgical interventions, insertion of artificial airways, mechanical ventilation, invasive lines, renal replacement therapies (continuous renal replacement therapy), tube feeding, noninvasive ventilation, and laboratory investigations.

The specific interventions outside the SICU such as magnetic resonance imaging (MRI), computed tomography (CT) scans, and ultrasound imaging were obtained from the PCI system as well. Blood and blood product use data were produced by the Pathology department on special request based on trauma patient medical records identification numbers.

Statistical analyses were performed using SPSS for Windows, Version 16.0 (SPSS Inc., Chicago, IL). The frequency and percentage were computed for qualitative observation and analyzed by chi-square test. The mean and standard deviation (SD) were estimated for quantitative observation and analyzed by t-test. The Mann-Whitney U-test was used for data that was not normally distributed. P ≤ 0.05 was considered statistically significant.

## Results

A total of 141 SICU admissions were reviewed for the six-month study period. Of those, 22.7% (32/141) were trauma patients. The average age of the trauma patients was 35 ± 13 years. The male-to-female ratio was 7:1 in the trauma cases. The overall mortality of the SICU was 39.7%, and the mortality for the trauma patients was 31.3%; the difference in mortality was minimal. The median length of stay was five days (interquartile range [IQR], eight days) as shown in Table 1.

**Table 1:** Comparison of outcomes between trauma and nontrauma patients (n = 141).

Variables	Trauma n = 32	Non-Trauma n = 109	P-Value
Age (Years)	36 ± 13	49 ± 19	0.0005
Gender (male/female)	28/4	72/37	0.019
	87.5%/12.5%	66.1%/33.9%	
Length of stay (days)	19.34 ± 14.09	17.43 ± 18.15	0.58
Outcome (Alive/Death)	22/10	63/46	0.26
	68.8%/31.3%	57.8%/42.2%	

**Figure 1:**
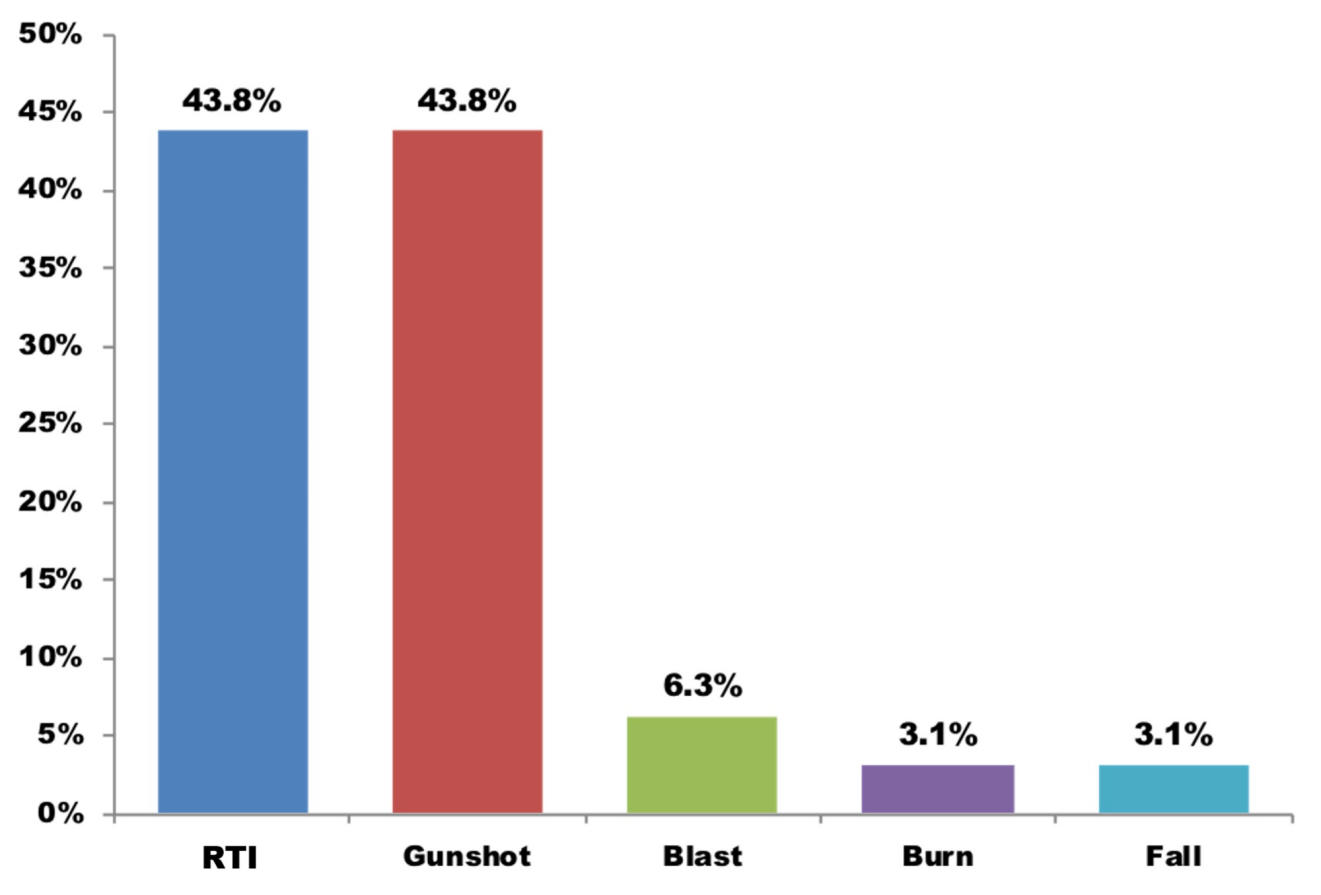
Type of trauma (n = 32). RTI: Road traffic injuries.

Figure 1 compares the incidence of the major types of trauma for admitted SICU patients. Gunshot and road traffic injuries were the most common reasons with equal incidences, followed by injuries from blasts, burns, and falls.

**Figure 2:**
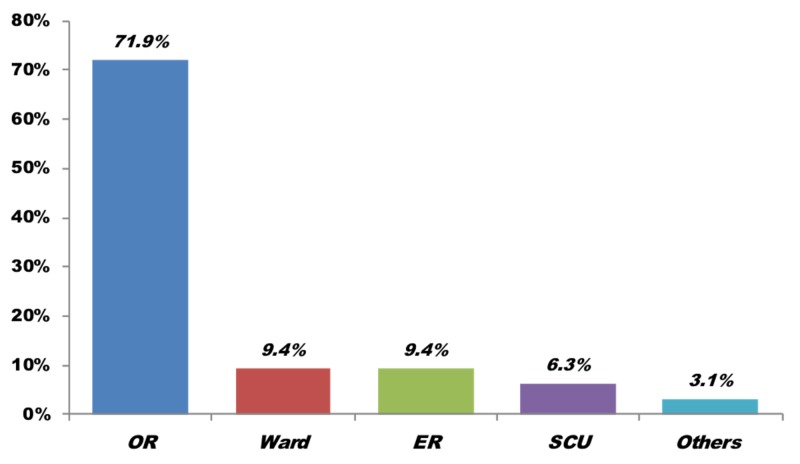
Sources of admission (n = 32). OR: Operating room; ER: Emergency room; SCU: Special care unit.

Figure 2 compares the sources of admission to the SICU. Most trauma patients in the SICU arrived from the operating room, while a smaller percentage of trauma patients arrived from the ward, emergency room, and special care unit (SCU).

Approximately 75% of all trauma patients underwent surgical interventions, whether before or after the admission, and only 3% of those were elective. The remaining 72% had emergency surgical procedures, which is a huge proportion and a major draw on resources.

**Figure 3:**
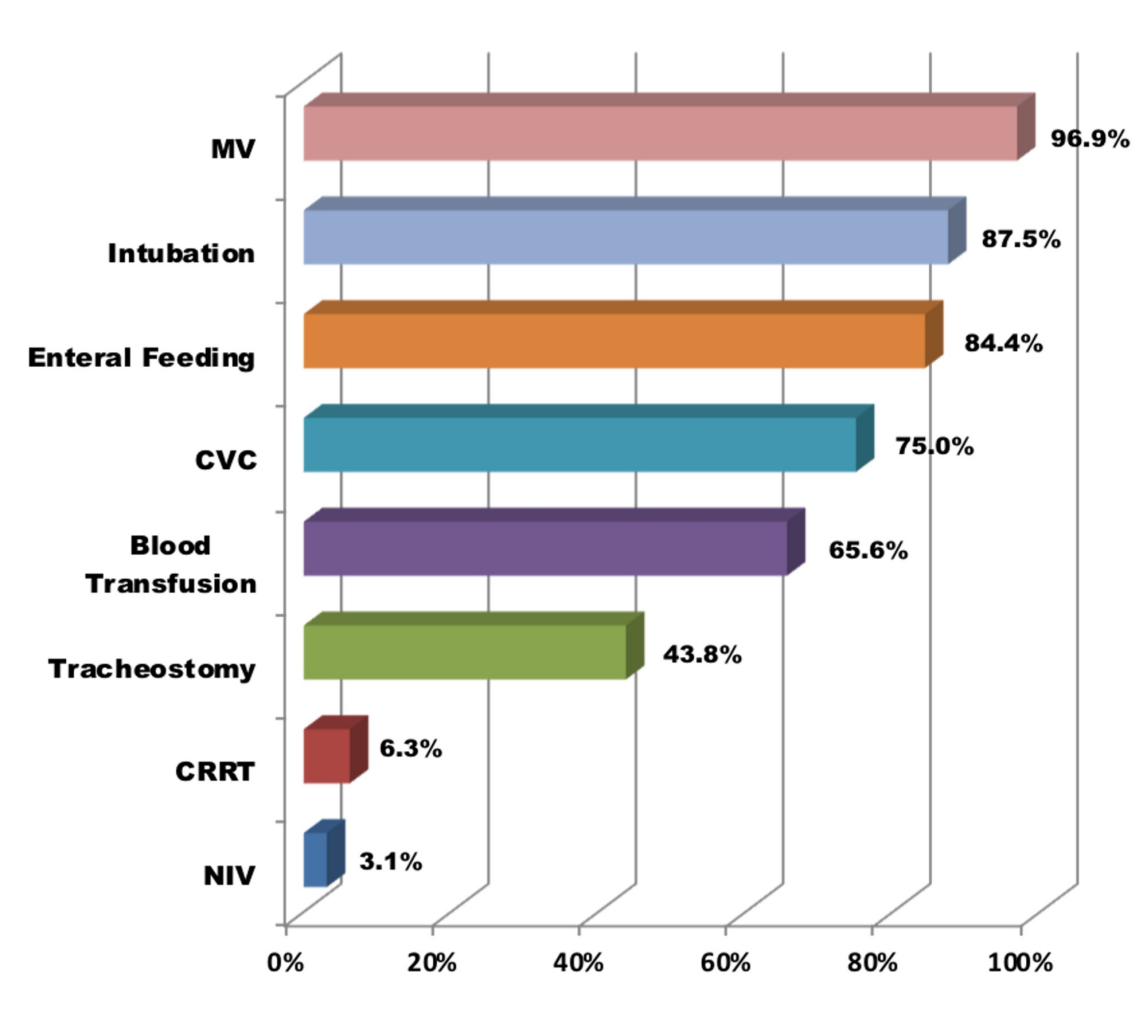
Therapeutic interventions in patients with trauma (n = 32). CVC: Central venous catheterization; CRRT: Continuous renal replacement therapy; NIV: Noninvasive ventilation; MV: Mechanical ventilation.

Figure 3 presents the percentage of nonsurgical therapeutic interventions for all trauma patients in the study. Mechanical ventilatory support was the most prominent (96.9%) followed by intubation (87.5%).

Table 2 presents the number of the patients who used blood products. The number of packed red blood cell (PRBC) units was the most commonly used blood product, and only two patients used a large amount of cryoprecipitate.

**Table 2:** Blood product use in trauma patients (n = 32). PRBC: Packed red blood cell; FFP: Fresh frozen plasma.

Blood product	Number of patients (%)	Median	Max-Min
PRBC	19 (59%)	11	79-1
FFP	11 (34%)	22	105-10
Platelets	12 (38%)	14.5	48-2
Cryoprecipitates	2 (6%)	57	100-54

Table 3 presents the number of advanced radiology investigations. CT scans were the most common advanced radiologic diagnostic tool for trauma patients.

**Table 3:** Major radiological procedures in trauma patients (n = 32). CT: Computed tomography; MRI: Magnetic resonance imaging; US: Ultrasonography; NA: Not applicable.

Radiology	Total	Number of Radiology/Patients			
		1	2	3	4
CT	31	9	9	9	4
MRI	5	4	1	NA	NA
US	19	11	8	NA	NA

The common laboratory investigations in terms of numbers of times performed are presented in Table 4.

**Table 4:** Frequency of laboratory investigations in trauma patients (n = 32). CBC: Complete blood count; BUN: Blood urea nitrogen; PT: Prothrombin time; APTT: Activated partial thromboplastin time; LFT: Liver function test; ABG: Arterial blood gas; CS: Cultures.

Test	Mean frequency of test performance (SD)	Minimum	Maximum
CBC	17.03 (13.87)	3	60
BUN	16.56 (14.85)	2	58
Creatinine	18.69 (17.17)	2	70
Electrolytes	21.44 (18.14)	3	72
PT (s)	11.75 (12.81)	1	51
APTT (s)	11.41 (12.80)	1	51
LFT	1.31 (2.28)	0	8
ABGs	18.13 (18.78)	1	77
Lactic acid	2.09 (2.41)	0	8
Procalcitonin	0.00 (0.00)	0	0
Blood CS	4.28 (4.62)	0	16
Tracheal CS	1.06 (2.14)	0	8
Urine CS	1.81 (1.75)	0	7

The electrolyte measurement test had the highest mean frequency (performed 21.44 times; SD, 18.14) followed by creatinine, arterial blood gas, complete blood count, and blood urea nitrogen.

## Discussion

The ICU is the main stem of every tertiary health care center where the sickest patients are given organ support and require a high degree of resources. We studied the use of resources by trauma patients in some detail in an SICU and found road traffic injuries (14/32) and gunshot wounds (14/32) were the two most common reasons for admission (comprising 87.5% of the workload). The average mortality was 39.7% in both trauma and non-trauma patients.

Abubakar et al. performed a similar study in a combined ICU of a developing country. Most ICU admissions in their study (90.3%) were related to surgical specialties, of which trauma cases (45.6%) were most common [2]. Their reported incidence was higher than ours (22.7%), and they had a 65% survival and 35% deaths with an overall mortality rate of 35%, which was similar to our findings.

Chalya et al. reported a higher incidence of trauma cases (37.1%) compared to our study but a similar male-to-female ratio of 5.5:1 compared to our 7:1. Road traffic crash was the most common cause of injuries in their study, affecting 70.8% of patients and of those, 68.6% required surgical intervention [3]. In our study, gunshot and road traffic had the highest incidence rate (43.8% each). We found similar mortality rates between trauma (31.3%) and nontrauma patients (42.2%; P = 0.26), whereas Chalya et al. reported trauma patients had a significantly higher mortality rate than that of all ICU admissions (32.7% vs. 18.8%, respectively; P = 0.0012) [3].

Radjou et al. found road traffic injuries caused the highest mortality (84%) which is a correlation of the patient admitted and type of injury. Our admitted trauma patients most consisted of road traffic injuries and gunshots wounds (87.5%) [4].

PRBC use was the most prominent in our study (59%). A study from a developed country with a large study population in an acute care inpatient setting reported 4805 (5.3%) patients received at least one unit of PRBCs [5]. However, blood and blood products should be used according to hospital guidelines [6].

While our radiological data may not have presented conclusive information, radiology use is a contributing factor in the overall burden on resources, especially given only trauma patients used these resources. One major study reported the overall rate of advanced imaging utilization decreased significantly from 172 per 100 ICU admissions in 2007 to 149 per 100 ICU admissions in 2011 (p = 0.012) [7]. The authors report the change was likely attributable to a major decrease in the use of CT (Poster Session, Epidemiology/Outcomes: Jarone L, Geyer B, Kaafarani H: Trends in Advanced Imaging Utilization in Intensive Care Units at Two Major Academic Hospitals. Critical Care Medicine, 2013, 41:12. 10.1097/01.ccm.0000439811.28707.44).

Lee and Maslove reported a decline in the expected amount of novel information provided by laboratory testing [7]. The Canadian Agency of Drug and Technologies for Health conducted a rapid response report for daily blood testing in the ICU from five nonrandomized studies (NRS). Three of the five NRS showed the number of routine laboratory tests decreased significantly, resulted in less anemia and substantial cost savings without compromising patient outcomes [8].

Given our study only used six months of data, we could not infer the utility of daily routine laboratory testing. However, the utility of laboratory testing is worth exploring when evaluating areas to reduce wasted resources.

We were unable to collect data on the cost of different variables, and our study period of six months was too brief to determine meaningful trends. Nevertheless, considering this first effort in our country, this study represents a meaningful opportunity to further explore and document new protocols and policies in our many hospitals.

## Conclusions

Trauma patients constitute a significant burden in terms of resources used for the SICU of Aga Khan University Hospital. Most trauma victims are young men suffering from either gunshot or road traffic injuries. Emergency surgical interventions are the largest burden on hospital resources, followed by blood product use, radiological resources, and laboratory investigation resources. By understanding the major sources of burden on hospital resources, important changes to policies and guidelines can be discussed by lawmakers and health care providers. Some changes, including the availability of firearms, vehicle safety precautions, driver skills, and traffic laws require input from policymakers outside of the health care system.

## References

[1] Chaira O, Cimbanissi S (2003). Organized trauma care: does volume matter and do trauma centers save lives?. Curr Opin Crit Care.

[2] Abubakar AS, Ojo EO, El-Nafaty AU, Edomwonyi NP (2007). An audit of one-year intensive care practice in a developing country. Int J Anesthesiol.

[3] Chalya PL, Gilyoma JM, Dass RM (2011). Trauma admissions to the Intensive care unit at a reference hospital in Northwestern Tanzania. Scand J Trauma Resusc Emerg Med.

[4] Radjou AN, Balliga DK, Pal R, Mahajan P (2012). Injury-related mortality audit in a regional trauma center at Puducherry, India. J Emerg Trauma Shock.

[5] Trentino KM, Farmer SL, Swain SG (2015). Increased hospital costs associated with red blood cell transfusion. Transfusion.

[6] Klein AA, Arnold P, Bingham RM (2016). AAGBI guidelines: the use of blood components and their alternatives 2016. Anaesthesia.

[7] Lee J, Maslove DM (2015). Using information theory to identify redundancy in common laboratory tests in the intensive care unit. BMC Med Inform Decis Mak.

[8] Canadian Agency for Drugs and Technologies in Health (2018). Routine blood tests for patients in the intensive care unit: clinical effectiveness, cost-effectiveness, and guidelines. CADTH.

